# The first giant cell arteritis hospital quality standards (GHOST)

**DOI:** 10.1038/s41433-023-02604-x

**Published:** 2023-06-21

**Authors:** Edward J. Bilton, Fiona Coath, Ajay Patil, Colin Jones, Eoin O’Sullivan, Melanie Hingorani, Chetan Mukhtyar, Susan P. Mollan

**Affiliations:** 1 Ophthalmology Department, Queen Elizabeth Hospital, University Hospitals Birmingham NHS Foundation Trust, Birmingham, B15 2TH UK; 2 INSIGHT Health Data Research Hub for Eye Health, University Hospitals Birmingham NHS Foundation Trust, Birmingham, B15 2TH UK; 3 Vasculitis Service, Rheumatology Department, Norfolk and Norwich Hospital, Colney Lane, Colney, Norwich, NR4 7UY UK; 4 Ophthalmology Department, Norfolk and Norwich Hospital, Colney Lane, Colney, Norwich, NR4 7UY UK; 5 Eye Unit, Kings College Hospital, London, SE5 9RS UK; 6 Moorfields Eye Hospital, City Road, London, EC1V 2PD UK; 7 Transitional Brain Science, Institute of Metabolism and Systems Research, College of Medical and Dental Sciences, University of Birmingham, Birmingham, B15 2TT UK

**Keywords:** Inflammatory diseases, Eye diseases, Diagnosis, Health services

Giant cell arteritis (GCA) remains a clinically challenging disease, despite advances in diagnostics and immunological therapies [1]. A significant concern is that people with GCA have the potential for sudden and devastating loss of vision. In a recent report from an interdisciplinary cohort of 350 consecutively diagnosed patients, the incidence of visual loss was 14% [2]. Mostly this occurs early in the disease, with few people developing sight loss on treatment or rarely during follow-up [1, 2]. In the United Kingdom (UK), the number of patients investigated for suspected GCA continues to grow [3]. Despite this growth, there are wide gaps in services, with more than a third of National Health Service (NHS) hospitals in England without a formal clinical pathway for GCA [4]. This raises concerns about the equity of access to NHS care for a serious common condition. It also has the potential to create disparity in outcomes, not only for those who are subsequently found to have GCA, but also for those people found to have serious alternative diagnoses [1].

Recent guidelines have made recommendations based on the advancing evidence for the investigation and management of GCA, and are commended to all healthcare professionals that deal with suspected GCA [5–7]. Although these recommendations exist, there was a need to develop quality standards for the management of GCA [8, 9].

Coath et al. [10] formed a multidisciplinary steering committee of rheumatologists, ophthalmologists, nursing staff and representation from the patient charity Polymyalgia Rheumatica and Giant Cell Arteritis UK (PMRGCA UK) to develop the first quality standards for the care of people with GCA. The aim was to define what aspects of clinical services are essential to provide excellence in GCA investigation and management and use this to create quality standards that can help to develop and benchmark services. The committee were asked to anonymously put forward up to five aspects of GCA services that they considered essential for the best clinical care. Common themes were identified, subsequently condensed into domain headings, and then ranked in order of importance. Quality standard statements for each domain were drafted and required a minimum 75% agreement to be accepted by the committee.

**Fig. 1:**
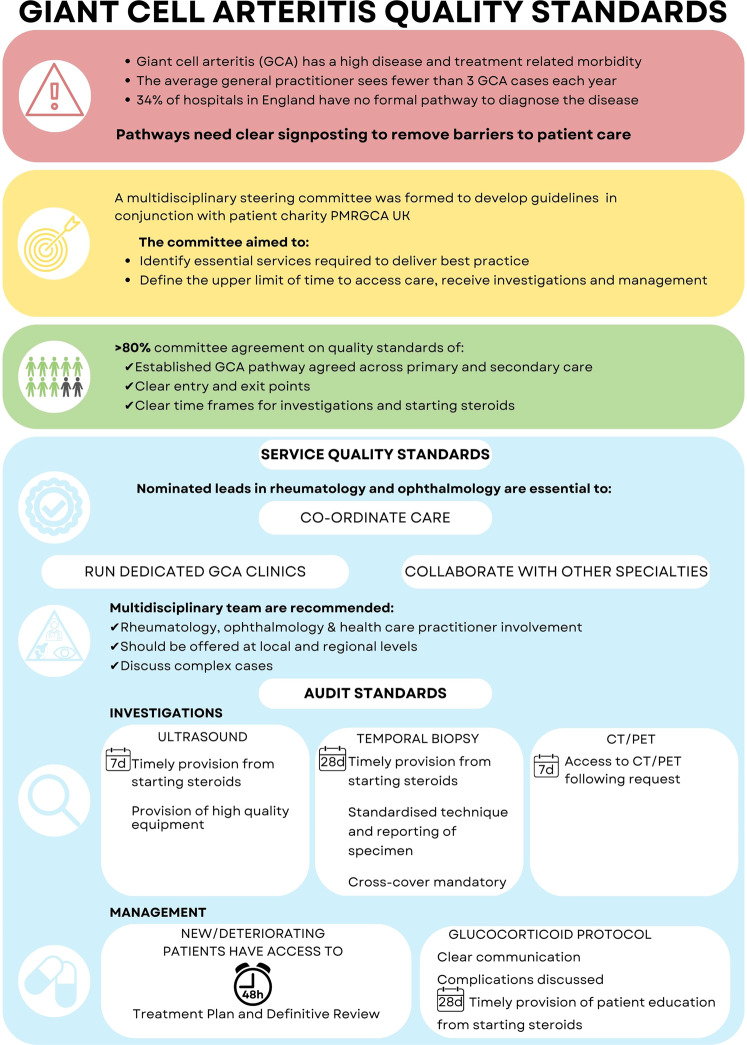
Infographic representing the Giant Cell Arteritis quality standards. Symbols used include: caution sign (current concerns); target sign (methods used); people ikon (compostiion of the committee); award and job type (service quality standards); magnifying glass (investigations) and pill ikon (depicts management).

This work is important to highlight to ophthalmologists as it defines key aspects of GCA care and recommends the ideal configuration of services, with time targets for investigation and management (Fig. 1). To ensure that care is coordinated and that there is a collaboration between specialties, they recommended nominated clinical leads in both rheumatology and ophthalmology. They agreed that multidisciplinary teams should exist to allow discussion of diagnostic dilemmas and review audit and morbidity data.

Auditable quality metrics that should be endorsed include maximum time frames for investigations with ultrasound, temporal artery biopsy and, when required, CT/PET imaging (Fig. 1). Management standards include access to specialist care within 2 working days and a definite treatment plan. They recommended all patients should be educated on the condition and complications of treatment (Fig. 1).

People with GCA are treated by many medical disciplines and there should be equity of access to high-quality clinical care throughout the UK. These GCA hospital standards (GHOST) have provided a quality framework for the improvement of pathways and services to be modeled upon [10].
